# Effect of Dehydroepiandrosterone Administration in Patients with Poor Ovarian Response According to the Bologna Criteria

**DOI:** 10.1371/journal.pone.0099858

**Published:** 2014-06-16

**Authors:** Bei Xu, Zhou Li, Jing Yue, Lei Jin, Yufeng Li, Jihui Ai, Hanwang Zhang, Guijin Zhu

**Affiliations:** Reproductive Medicine Center, Tongji Hospital, Tongji Medicine College, Huazhong University of Science and Technology, Wuhan, People's Republic of China; University of Kansas Medical Center, United States of America

## Abstract

**Background:**

Dehydroepiandrosterone (DHEA) is now widely used as an adjuvant to IVF treatment protocols in poor responders. However, clinical evidence for DHEA on improvement of ovarian response and IVF outcome is still limited, the validity of the results of the earlier studies, especially the varied inclusion criteria, is a subject of debate. Recently, the ESHRE Working Group developed a new definition, the Bologna criteria. The aim of the current study was to investigate the potential effect of DHEA treatment on in vitro fertilization (IVF) outcome of poor ovarian responders that fulfill the Bologna criteria.

**Methods:**

This study investigated 386 poor ovarian responders that fulfill the Bologna criteria. Patients underwent IVF-ET treatment with the GnRH antagonist protocol. The study group contained 189 patients, who received 75 mg of DHEA daily (25 mg three times daily) before the IVF cycle. The control group was composed of 197 patients who received infertility treatment, but did not receive DHEA. The IVF outcome parameters in each group were compared.

**Results:**

The study and control groups did not show statistically significant differences in terms of patient demographics characteristics, mean numbers of oocytes retrieved, mature oocytes, fertilization rate, cleavage rate, or embryo availability. While the DHEA group demonstrated significantly higher implantation rates (18.7% vs. 10.1%; *P*<0.01) and ongoing PRs (26.7% vs. 15.8%; *P*<0.05) as compared with the control.

**Conclusions:**

DHEA pre-treatment does not significantly increase oocyte yield. However, the ongoing PRs in this subgroup of women are significantly higher after DHEA administration, suggesting that DHEA may increase IVF results by improving oocyte and embryo quality.

## Introduction

Poor ovarian response represents one of the few unresolved problems of modern infertility care. The overall incidence of poor ovarian response has been reported to be between 9 and 24% [1].

A variety of regimens have been employed, including the use of increased gonadotropin doses, low dose gonadotropin-releasing hormone (GnRH) agonists or antagonists, flare regimes, adjunctive growth hormone, minimal ovarian stimulation with clomiphene citrate, and natural cycle IVF [2], [3]. However, the ideal stimulation regimen for poor responders is currently unknown. In addition, there is insufficient evidence to support the use of specific interventions to improve IVF treatment outcomes in poor responders. The Cochrane review of poor responder interventions concluded that no particular treatment offered clear benefit, or could be recommended [4]. The management of poor responders therefore remains a significant challenge in assisted reproduction.

In the last few years, a number of studies have suggested that dehydroepiandrosterone (DHEA) treatment may be effective in poor responders [5]–[9]. DHEA is now widely used in poor responders; recently, a world-wide survey showed that 26% of IVF clinicians in 45 countries add DHEA as an adjuvant to IVF treatment protocols in women with poor ovarian response [10]. However, clinical evidence for DHEA on improvement of ovarian response and IVF outcome is still limited, the validity of the results of the earlier studies, especially the varied inclusion criteria, is a subject of debate [11]. Recently, the Consensus Group for the European Society for Human Reproduction and Embryology (ESHRE) developed a new definition, the Bologna criteria, to help assigning more uniform patient groups in future clinical trials [12]. However, no studies have been performed study to evaluate the potential effects of DHEA supplementation according to these standards.

We therefore decided to assess the impact of DHEA supplementation on IVF outcome of poor ovarian responders that fulfill the Bologna criteria.

## Materials and Methods

### Study Design

This was a retrospective cohort study. Patients were treated at Tongji Hospital between October 2011 and July 2013. The study conformed to the “Declaration of Helsinki for Medical Research involving Human Subjects”. Also, approval was obtained from the Ethical Committee of the Tongji Medical College. Each of the patients had given written authorization at the time of treatment for the future use of their clinical data.

### Study Participants

A total of 386 patients undergoing IVF/ICSI treatment were enrolled. Of these, 90 women have undergone only one IVF cycle, and 296 women were diagnosed with POR in their preceding IVF cycle. All patients were stratified according to the Bologna criteria for poor ovarian response. Poor responders were classified with at least two of the three following criteria: (I) advanced maternal age (≥40 years) or any other risk factor for POR; (II) a previous POR (≤3 oocytes with a conventional ovarian stimulation protocol); and (III) an abnormal ovarian reserve test (ORT): antral follicle count (AFC) <5, as described by the Bologna criteria [12]. Excluded from the study were women aged >45 years or baseline FSH levels >40 IU/l. All patients were thoroughly informed about the novelty and unknown efficacy of DHEA in improving ovarian response. The patients of study group received DHEA 25 mg orally,three times a day before the IVF cycle. Except for IVF, the control group of patients did not receive any pre-treatment.

### Protocol for COH

Patients underwent controlled ovarian hyperstimulation (COH) with the use of a GnRH antagonist protocol. COH was carried out in an identical manner in the two groups, with 150–300 IU of recombinant FSH (Gonal-F,Merck-Serono, Switzerland) and 75–225 IU of highly purified urinary gonadotropine (hMG, Lizhu, China), from day 2 or 3 of the cycle, according to age, basal FSH levels, antral follicle count (AFC), and previous response to gonadotropins. Daily injections of a GnRH antagonist (CetrotideR 0.25 mg sc, Merck-Serono, Switzerland) were administered to prevent premature ovulation by using the flexible antagonist protocol, according to a personalized regimen: From the day the leading follicle reached 14 mm in diameter until the day of hCG injection. Recombinant hCG (250 mg; Ovidrel; Serono) was given to trigger ovulation when two leading follicles reached a mean diameter of 18 mm. Oocytes were retrieved transvaginally 36–37 hrs after hCG administration. IVF or ICSI were performed as appropriate, with taking semen quality into account. Fewer than three embryos were transferred on day 2 or 3 after oocyte retrieval, excess good-quality embryos were cultured to the blastocyst stage and then cryopreserved for subsequent frozen embryo transfer (FET) cycles. The luteal phase was supported with 60 mg Progesterone injections IM from the day of oocyte retrieval. A quantitative pregnancy test (serum β-hCG based) was taken 14 days after hCG administration. In case of pregnancy, a transvaginal ultrasound was performed 28 days after the embryo transfer and repeated as required. Clinical pregnancy was confirmed if a fetal heartbeat was observed by transvaginal ultrasound.

### Hormone Measurement

Serum P and E2 levels were measured on the day of hCG administration. Samples were tested with a microparticle enzyme immunoassay (Axsym System, Advia Centaur; Siemens, Germany), which had a sensitivity of 0.21 ng/mL. Intra- and interassay coefficients of variability were 7.2% and 5.7%, respectively, for P. The E2 assay had a sensitivity of 7.0 pg/mL, with intra- and interassay coefficients of variability of 11.3% and 5.0%, respectively.

### Main measurement and outcomes

The primary outcome measure was ongoing PR and implantation rate. Ongoing pregnancy was defined as a pregnancy with a positive heartbeat by ultrasound after 12 weeks of gestation. The implantation rate was calculated by dividing the total number of fetal cardiac activities detected by the total number of transferred embryos. Cancellation rate was calculated as the number of cycles in which patients stopped treatment due to lack of embryos available for transfer divided by the total number of initial cycles. Other analyzed variables included the mean number of follicles ≥14 mm, mean serum oestradiol, and progesterone concentration on the day of hCG administration, mean number of retrieved oocytes, mean number of mature oocytes (MII), mean number of fertilized oocytes, cleavage embryos, embryos available, blastocysts, and of transferred embryos. In addition, mean cumulative gonadotrophin dose and duration of ovarian stimulation were also compared between the two groups.

### Statistical Analysis

All statistical calculations were performed using SPSS 15.0 software (IBM, New York, NY). Continuous variables were presented as mean value ± SD. Associations between demographic and clinical characteristics of the patients were assessed with the use of Student's t test or the Mann-Whitney U test for continuous variables, and the chi-square test for categorical variables, as appropriate. *P*<0.05 was considered to be statistically significant.

## Results

A total of 386 patients met the inclusion criteria. Among these were 212 women ≥40 years old. 23 patients had undergone ovarian cystectomy, 7 patients had only one ovary due to ovariectomy. 296 women presented after repeated IVF failures that were attributed to poor ovarian response.

DHEA group consisted of 189 patients and control group consisted of 197 patients. The study patients took DHEA for an average of 90 days before entry into their IVF cycles. The patient characteristics and basal ovarian reserve assessment were similar between all groups. There were no differences between the two groups regarding age, body mass index (BMI), duration of infertility, basal FSH, primary or secondary infertility, and antral follicle count (AFC) (see Table 1).

**Table 1:** Patient characteristics and basal ovarian reserve assessment in the DHEA and control groups.

Parameter	DHEA group	control group	P value
Number of cycles	189	197	
Number of ICSI cycles	46	44	
Cause of infertility(n)			
Tubal	120	126	
Unexplained	24	18	
Endometriosis	19	22	
Male factor	43	50	
Days of DHEA	90. 81±39.44	0	
Age (years)	37.67±4.67	38.1±4.22	N.S
BMI	21.77±5.9	21.39±5.1	N.S
Basal FSH (IU/1)	12.34±3.79	11.84±3.64	N.S
AFC	4.36±1.34	4.39±1.34	N.S
Primary infertility	67	70	N.S
Secondary infertility	122	127	N.S
Duration of infertility (years)	7.32±4.82	7.42±4.94	N.S

Note: Values are expressed as mean ± SD. NS  =  not statistically significant.

Paired IVF cycle characteristics between DHEA and control group are summarized in Table 2. The results show there are no differences in cycle cancellation rate, plasma E2 and P values on hCG day, duration of stimulation, endometrial thickness and the numbers of embryo transfer. The mean numbers of follicles ≥14 mm was significantly higher among patients after DHEA administration (*P*<0.05), while the amount of FSH required for COH was significantly decreased in the DHEA group (*P*<0.01). There was a trend towards mean numbers of oocytes retrieved, mature oocytes, embryos available, blastocysts, mean numbers of fertilized oocytes and cleavage embryos, fertilization rate, and cleavage rate in the DHEA group, but these failed to reach significance (*P*>0.05). However, there were significantly higher implantation rates in the DHEA group than in the control group, 18.7% versus 10.1%, respectively (*P*<0.01). Also ongoing PRs were higher in the DHEA group, 26.7% versus 15.8%, respectively (*P*<0.05) (see Table 3).

**Table 2:** Clinical features and ovarian stimulation outcomes in the DHEA and control groups.

Parameter	DHEA group	control group	P value
Number of FSH vials (IU)	37.4±11.3	41.49±11.05	0.007
Duration of stimulation (days)	10.19±2.00	10.14±1.97	N.S
Peak E2 value (pg/ml)	1501±696	1494±709	N.S
P value on hCG day (ng/ml)	1.13±0.43	0.96±0.47	N.S
Endometrial thickness on hCG day	9.41±1.78	9.85±1.86	N.S
Number of follicles≥14 mm	4.41±2.02	3.92±1.58	0.03
Number of oocytes retrieved	4.48±2.46	3.95±2.09	N.S
Number of mature oocytes	3.75±2.22	3.5±1.87	N.S
Number of fertilized oocytes	3.30±1.99	2.83±1.78	N.S
Number of cleavage embryos	3.23±1.99	2.76±1.75	N.S
Fertilization rate (%)	77.2±22.3	73.8±25.5	N.S
Cleavage rate (%)	97.3±5.22	96.3±6.87	N.S
Number of available embryos	1.97±1.35	1.80±1.24	N.S
Number of embryo transfer	1.34±0.86	1.36±0.90	N.S
Number of blastocysts	1.44±1.30	1.18±1.09	N.S

Note: Values are expressed as mean ± SD or percentage. Fertilization rate: two-pronuclear zygotes (2PNs)/total MII oocytes in ICSI cycle or fertilized oocytes/total oocytes in IVF cycle. Cleavage rate: embryos on the 3rd day of cleavage–stage/total fertilized oocytes.

**Table 3:** IVF cycle results in the DHEA and control groups.

Parameter	DHEA group	control group	P value
Cycle cancellation (%)	16.4	20.3	N.S
Implantation rate (%)	18.7	10.1	0.005
Clinical pregnancy rate (%)	30.4	18.7	0.025
Ongoing pregnancy rate (%)	26.7	15.8	0.028

Note: Values are expressed as percentage. Cycle cancellation rate: total number of cancellation/total initiated cycles. Implantation rate: total number of gestational sac/total embryos transferred; Clinical pregnancy rate: total number of clinical pregnancy/total transferred cycles; Ongoing pregnancy rate: total number of clinical pregnancy after 12 weeks of gestation/total transferred cycles.

The medication was well tolerated by all patients. No patient dropped out of treatment because of side effects attributed to DHEA use. Indeed, several patients reported to feel energized and have an increased feeling of wellbeing while using DHEA.

## Discussion

This study is the first to examine the effect of DHEA in poor ovarian responder patients that fulfill the Bologna criteria. According to our results, although the oocyte and embryo numbers were similar between the two groups, DHEA administration was associated with significantly higher implantation and ongoing PRs.

DHEA is a mild androgen, produced as an intermediate step by the adrenal glands (85%) and ovaries (15%) during steroidogenesis. The circulating levels of DHEA decline markedly with advancing age [13]. It is a very important prehormone for sex steroidogenesis. In the ovarian follicle, DHEA is converted to androstenedione and estrone, the source of testosterone and estradiol according to the two-cell theory [14]. Casson first speculated that exogenous administration of DHEA could restore ovarian follicular sex steroidogenesis in elderly women [5]. Since then, a few controlled studies with small sample sizes have reported the benefits of DHEA administration in improving ovarian response and IVF outcome [6]–[9], [15]–[17]. However, since women with significant diminished ovarian reserve usually are reluctant to enter prospectively randomized studies that may assign them to placebo, because of the limited time remaining to conceive with use of their own oocytes [18], nearly all DHEA data represented lower levels of evidence. The first RCT study with small sample size regarding DHEA administration before IVF in poor responders was conducted by Wiser and associates [9], who showed a significantly higher cumulative live birth rate among the DHEA group. In contrast, another recent RCT study with larger sample size failed to show that DHEA supplementation enhances IVF-ICSI outcome in women with poor ovarian reserve [20].

The meta-analysis which evaluated the effect of adjuvant androgens (DHEA or Testosterone) or androgen-modulating agents (Letrozole, aromatase inhibitors) in previous poor responders has failed to demonstrate any significant difference in the ongoing PRs, live birth rates, and numbers of oocytes retrieved when compared with the control groups[19]. Another systematic review by Bosdou *et al.* reports that only transdermal testosterone, but no other androgen modulating agents including DHEA achieved significantly improved clinical PRs [21]. Recently, a systematic review and meta-analysis that reported specifically on the role of DHEA alone in women with diminished ovarian reserve, suggested that DHEA does not improve the ovarian response and pregnancy outcome [22]. However, all these review are limited by small sample sizes.The review by Bosdou *et al.* on DHEA included only one study with 33 participants. Especially,the heterogeneity between the former studies is criticized, caused by the wide diversity in the definitions used to specify women with impaired response to ovarian stimulation [23].

Recently, a consensus was reached by the ESHRE Working Group on the criteria needed to define POR when at least two of the following three features must be present: (i) advanced maternal age or any other risk factor for POR; (ii) a previous POR and (iii) an abnormal ovarian reserve test (ORT) [12]. One of the most important components in the Bologna criteria is a previous POR and, therefore, one stimulated cycle is considered essential for the diagnosis of POR. However, patients over 40 years of age with an abnormal ORT may also be classified as poor responders since both advanced age and an abnormal ORT could indicate reduced ovarian reserve and act as a surrogate of ovarian stimulation cycle. Although the Bologna criteria have also been criticized for several limitations, such as the risk factors for POR were not clearly defined [24]. However, it is the only internationally accepted universal definition of POR, so that it is amenable for use in future clinical trials. Here we presented DHEA data by using these uniform inclusion criteria. The results showed significantly higher implantation rates and ongoing PRs were noted in the DHEA group, although there were no obvious beneficial effects of DHEA on oocytes yield in poor responders. These findings imply that DHEA may exert its positive effect by improving oocytes and embryo quality. In addition, our study shows that DHEA administration resulted in a significant increase in the total number of follicles that develop to a size ≥14 mm in response to FSH stimulation, while the total FSH dose required was significantly decreased in DHEA group. These suggest that exogenous DHEA increases the ovarian sensitivity to gonadotrophin stimulation in poor responders.

The mechanism of DHEA action on the ovaries remains speculative. Numerous hypotheses have been proposed on how DHEA may enhance fertility. One mechanism that has been suggested is a direct effect of DHEA on ovarian folliculogenesis by increasing primordial follicle pool up to the pre-antral and antral follicle stages [25]–[29]. Androgens may act on ovarian follicular development by increasing the number of FSH receptors expressed in the granulosa cells [30]–[33], and the increasing intrafollicular androgens could augment granulose cell anti-Müllerian hormone (AMH) production [15], [34], thus stimulating early stages of follicular growth [25], [35], [36]. It was recently proven in an animal experimental model that DHEA exposure stimulated initiation of primordial follicles and development of gonadotrophin-responsive preantral and early antral follicles through promoting granulosa cell proliferation [26]. Besides, oral DHEA administration has been demonstrated to increase serum IGF-I concentrations [5], which are known to have a positive effect on follicular development and oocyte quality. Further, DHEA may enhance the follicular microenvironment through reducing apoptosis of the originally recruited follicles and beneficially affecting mitochondrial function in both follicular cells and oocytes [25], [37]. In addition, given the published report of DHEA on reducing miscarriage rates in older patients [38], which may be attributed to diminished aneuploid embryo rates [39], DHEA may improve oocyte quality through the promotion of DNA repair in oocytes [40].

Women using DHEA may experience possible adverse effects including acne, hair loss, deepening of the voice, and facial hair growth. In this study, the DHEA dose of 75 mg/day was well tolerated by all patients. We did not encounter a single complication of clinical significance.

In summary, this is the first study to assess the effect of DHEA administration in poor ovarian responders as described by the Bologna criteria. DHEA pre-treatment in a GnRH antagonist setting does not appear to significantly increase oocytes yield; however, the ongoing PRs in this group are significantly higher after treatment of DHEA, suggesting that DHEA may increase IVF results by improving oocyte and embryo quality. We realize that the retrospective design could represent a weakness of our study; nonetheless, taking into account that we included only consecutive patients that fulfill the Bologna criteria and were treated with exactly the same protocol, the likelihood of selection bias might be limited. Additional larger, multicenter, randomized trials are needed to extend and reinforce our findings.
